# Mechanisms of Cong Rong Shu Jing Compound Effects on Endoplasmic Reticulum Stress in a Rat Model of Parkinson's Disease

**DOI:** 10.1155/2020/1818307

**Published:** 2020-05-14

**Authors:** Yao Lin, Lanfang Tang, Peizhen Huang, Ting Liu, Jianan Zhong, Qian Xu, Xiyu Li, Youning Lin, Shaojian Xiao, Jing Cai

**Affiliations:** ^1^College of Integrative Medicine, Fujian University of Traditional Chinese Medicine, Fuzhou 350122, China; ^2^Academy of Integrative Medicine, Fujian University of Traditional Chinese Medicine, Fuzhou 350122, China; ^3^Third People's Hospital, Fujian University of Traditional Chinese Medicine, Fuzhou 350122, China

## Abstract

This study investigated the effects of the Cong Rong Shu Jing (CRSJ) compound on endoplasmic reticulum stress in a rat model of Parkinson's disease (PD). A total of 40 rats were subcutaneously injected with rotenone-sunflower oil emulsion into the back of the neck to establish a rat model of PD. These PD rats were randomly divided into low-, medium-, and high-dose groups (intragastric administration of 0.5, 1, and 2 g/kg CRSJ, respectively) and a model group (intragastric administration of the solvent; 10 rats per group). Furthermore, 10 rats each were attributed to the control and vehicle groups (both received intragastric administration of the CRSJ solvent, and the vehicle group were injected additionally with sunflower oil alone). A traction test was conducted two times, after the PD model establishment and after 14 days of CRSJ gavage. The numbers of tyrosine hydroxylase- (TH-) positive cells and the dopamine levels in the substantia nigra were assessed using immunohistochemistry and high-performance liquid chromatography, respectively. Western blotting detected the expression levels of *α*-synuclein, endoplasmic reticulum stress pathways-related proteins, cerebral dopamine neurotrophic factor (CDNF), mesencephalic astrocyte-derived neurotrophic factor (MANF), and phosphoinositide 3-kinase (PI3K)/protein kinase B (AKT) pathway-related proteins. Compared with the model group, the number of TH-positive cells in the substantia nigra was increased in the CRSJ groups. The expression levels of *α*-synuclein and the endoplasmic reticulum stress pathways-associated proteins glucose regulatory protein 78, inositol-requiring enzyme 1, apoptosis signal-regulating kinase 1, phosphorylated c-Jun N-terminal kinase, and caspase-12 were reduced. However, CRSJ administration elevated the expression levels of the neurotrophic factors CDNF and MANF, as well as those of p-PI3K and p-AKT. The CRSJ compound can relieve endoplasmic reticulum stress in PD rats and exerts protective effects in this animal model. These effects may be related to increased expression of neurotrophic factors and activation of the PI3K/AKT pathway.

## 1. Introduction

Typical pathological manifestations of Parkinson's disease (PD) are the degeneration and loss of neurons specifically in the dense part of the substantia nigra, as well as the formation of Lewy bodies in the residual neurons. Lewy bodies are mainly composed of a large number of un- or misfolded *α*-synuclein proteins, and their abnormal aggregation or precipitation can cause endoplasmic reticulum stress (ERS), which ultimately induces the degeneration of dopaminergic neurons [1]. Glucose regulatory protein 78 (GRP78) is a molecular chaperone of the endoplasmic reticulum. It binds to membrane proteins of the endoplasmic reticulum, such as inositol-requiring enzyme 1 (IRE1*α*), thus preventing ERS. But when abnormal proteins such as *α*-synuclein accumulate intracellularly, they can cause the disintegration of transmembrane proteins and initiate ERS. ERS not only facilitates the correct folding and translocation of *α*-synuclein but also activates IRE1*α*, which activates downstream a series of enzymes, such as apoptosis signal-regulating kinase 1 (ASK1), c-Jun N-terminal kinase (JNK), and caspase-12, among others, ultimately leading to neuronal apoptosis [2].

Our research group is experienced in studying the effects of the Cong Rong Shu Jing (CRSJ) compound, and we have applied for its national patent. Previous studies of our group have shown that the main components of *Cistanche* spp. and *Polygonatum* spp. can improve the Unified Parkinson's Disease Rating Scale (UPDRS) score of patients with Parkinson's disease [3], increase the expression of neurotrophic factors in the brain of PD model rats [4], and enhance the expression of phosphoinositide 3-kinase (PI3K) and protein kinase B (AKT) [5, 6]. Some studies have shown that neurotrophic factors can activate the PI3K/AKT pathway, thereby inhibiting ERS and reducing nerve cell apoptosis [7]. Whether CRSJ can alleviate neuronal apoptosis by regulating the GRP78-IRE1*α*-ASK1-JNK signaling pathway related to ERS and whether these ERS-regulating mechanisms are involved in the increased expression of neurotrophic factors and activation of the PI3K/AKT pathway still needs to be confirmed by experiments. We conducted the following research to explore the CRSJ-induced mechanisms in the treatment of PD.

## 2. Materials and Methods

### 2.1. Animals, Reagents, and Equipment

A total of 70 healthy male Sprague-Dawley rats (specific-pathogen-free grade, weight 180 ± 20 g) were provided by the Experimental Animal Center of the Fujian University of Traditional Chinese Medicine. The animals were kept under the following conditions: room temperature, 20–22°C; relative humidity, 60–65%; automatic light-dark control (light from 7 : 00 to 19 : 00, dark from 19 : 00 to 7 : 00); and food and water ad libitum.

CRSJ granules, composed of *Cistanche deserticola*, *Polygonatum* spp.*, Salvia miltiorrhiza*, red peony root, and peony skin, were provided by the Third People's Hospital Affiliated to Fujian University of Traditional Chinese Medicine. Additionally, the following drugs were used in this study: rotenone (no. R8875) and sunflower oil (no. S5007; both Sigma-Aldrich, St. Louis, MO, USA); urethane (no. S11036; Shanghai Yuanyi Biotechnology Co., Ltd., China); immunohistochemical kit (no. kit-9720; Fujian Maixin Technology Co., Ltd., China); ECL chemiluminescence detection kit (no. 1705060; Bio-Rad, Hercules, CA, USA); BCA kit (no. P0010S; Shanghai Biyuntian Institute of Biotechnology, China); dopamine (DA) standard (no.161136; Sigma-Aldrich, Carlsbad, CA, USA); cerebral dopamine neurotrophic factor (CDNF) antibody (no. NBP1-76834; Novus Biologicals, Littleton, CO, USA); PI3K antibody (no. 4257), AKT antibody (no. 9272), phosphorylated (p)-PI3K antibody (no. 4228), p-AKT antibody (no. 4060), and p-JNK antibody (no. 81E11; all Cell Signaling Technology, Beverly, MA, USA); beta-actin antibody (no. 66009–1; Wuhan Sanying Company); and mesencephalic astrocyte-derived neurotrophic factor (MANF) antibody (no. Ab67271), tyrosine hydroxylase (TH) antibody (no. Ab112), GRP78 antibody (no. Ab108613), ASK1 antibody (no. Ab131506), JNK antibody (no. Ab179461), and caspase-12 antibody (no. Ab62484; all Abcam, Cambridge, MA, USA).

Furthermore, the following equipment was utilized: inverted microscope (TS100; Nikon, Tokyo, Japan); brightfield microscope (MDL) and microtome (RM2235; both Leica, Wetzlar, Germany); high-performance liquid chromatography (HPLC; Agilent 1200; Agilent Technologies, Santa Clara, CA, USA); microplate reader (ELX800; BioTek, BioTek Winooski, Vermont, USA); and electrophoresis apparatus (PowerPac Basic), transfer film tester (PowerPac Basic), and a chemiluminescence gel documentation system (Universal Hood II; all Bio-Rad).

### 2.2. Establishment of the Animal Model

The PD model was prepared according to the classic method established by Sherer et al. [8]. Briefly, rotenone was dissolved in sunflower oil emulsion at a final concentration of 1.5 mg/ml. Rats were subcutaneously injected into the neck and back with 1.5 mg/(kg·d) rotenone for 14 consecutive days. The behavioral changes of the rats were observed and scored according to the behavioral scoring standard of Chen et al. [9]. Animals with scores between 2 and 8 qualified as PD model rats.

### 2.3. Grouping and Drug Administration

According to their random number generated by the SPSS 24.0 software, 70 healthy rats were divided into the control group (10 rats, no treatment), the vehicle group (10 rats, injection of the same amount of sunflower oil emulsion without rotenone into the neck and back of the animals to exclude any influence of the solvent), and the model group (50 rats, rotenone injections). After the 14-day period to induce PD in the animals, 3 and 7 rats had behavioral scores of 1 point and 10 points, respectively. They were excluded, leaving a total of 40 rats with PD for further experiments. These successfully modeled animals were randomly divided into the model group and the low-, medium-, and high-dose CRSJ compound groups with 10 rats in each group.

According to the established human daily CRSJ dosage of 10 g per person and the known body-surface area ratio of man to rat [10], the low, medium, and high doses of daily medication in rats were calculated as 0.5, 1, and 2 g/kg, respectively. The medium dose of 1 g/kg was the human-equivalent dose in rats. Immediately after confirming PD in the animals, they were treated once a day for 14 consecutive days with either different CRSJ doses or an equivalent saline volume. The low-, medium- and high-dose groups received intragastric administration of 50, 100, and 200 mg/ml CRSJ, respectively, in a final volume of 10 ml/kg.

### 2.4. Behavioral Test

The traction test was performed in all rats twice, first after the establishment of the PD model and again after the two weeks of CRSJ administration [11]. The rats were hanging from a horizontal wire 30 cm above the ground, and the latency to the time when the animals landed on the ground was scored according to the following criteria: 0–5 s, 0 points; 6–10 s, 1; 11–15 s, 2; 16–20 s, 3; 21–25 s, 4; 26–30 s, 5; and >30 s, 6 points. The test was repeated three times with a rest of 2 min between trials, and the average was calculated.

### 2.5. Specimen Collection

Brain tissue samples were collected in all groups after the second traction test had been conducted following the 14-day CRSJ administration period. From each group, three rats were anesthetized with an intraperitoneal injection of 5 ml/kg 20% urethane. The chest was opened, an infusion needle was inserted into the left ventricle, and the right auricle was cut. The animal was perfused with physiological saline at 4°C while observing the liver for bleaching as a sign of perfusion success. After the outflowing fluid of the right atrium became transparent, animals were completely transfused with a 4% paraformaldehyde solution before carefully removing the brain. The brain tissue was fixed in 4% paraformaldehyde solution for 24 h, rinsed for 2 h with water, and dehydrated with a 70%, 80%, 90%, 95%, and 100% ethanol gradient. The tissue blocks were transparentized with xylene, embedded in paraffin wax, and sliced for immunohistochemical experiments. The remaining seven rats per group were completely anesthetized, their heads were cut off, and their brains were removed immediately and kept in an ice-cold solution. A tissue block containing the striatum (1 mm in front of the anterior fontanelle, 3 mm near the median line, and 4.5 mm below the dura) was removed, and the tissue was repeatedly rinsed with ice-cold 0.9% sodium chloride solution to remove the blood. The tissue was dried with filter paper and stored in a freezer at −80°C for later use.

### 2.6. TH Expression Measured by Immunocytochemistry

The paraffin sections were immunohistochemically stained according to the kit instructions and observed using a 100× and 400× microscope. Brown- or light-yellow particle stains were considered positive for TH expression. Five fields (100×) were randomly selected from each section, their images were analyzed using the Image-Pro Plus 6.0 software, the number of TH-positive cells was determined, and the average was calculated.

### 2.7. Detection of DA in the Striatum by HPLC

The striatum specimens of each group of rats were homogenized in 0.4 mol/l HClO_4_ and then centrifuged at 4°C for 15 min at 12000 r/min. The supernatants were collected, the protein concentration was detected using the BCA method, and the dopamine content was detected by HPLC.

### 2.8. Western Blotting Analysis In Vivo

Each rat striatum was weighed, and lysis buffer was added for a lysate concentration of 12.5 *µ*l/mg. The samples were fully homogenized, centrifuged at 4°C at 12000 r/min for 15 min, the supernatants were collected, and the protein concentrations were determined using the BCA method. The sample was heated in boiling water for 10 min to fully denature the proteins. Solutions containing antibodies against *α*-synuclein, GRP78, IRE1*α*, p-IRE1*α*, ASK1, JNK, p-JNK, caspase-12, CDNF, MANF, PI3K, p-PI3K, AKT, or p-AKT at 1 : 1000 were added and kept overnight at 4°C. The next day, the membranes were washed with TBST before adding the secondary antibody at 1 : 2000. These samples were incubated at room temperature for 1 h, and then the membranes were washed again. To detect the antibodies, the ECL chromogenic agent (chemiluminescence substrate and stabilizer, 1 : 1) was added and allowed to react for 1 min. The blot image was taken afterward in the Universal Hood II, and the Image Lab 4.0 software was employed to analyze the grayscale values of the protein bands, using beta-actin levels as the internal reference. The target protein bands are expressed as the ratio target protein expression/beta-actin expression.

### 2.9. Statistical Methods

SPSS 24.0 software was used for statistical processing, and data are presented as the mean ± standard deviation. One-way analysis of variance was conducted for comparisons between groups, and groups were pairwise compared using Fisher's least significant difference or Games-Howell post hoc tests. The test level was set at 0.05.

## 3. Results

### 3.1. Protective Effects of CRSJ in the Rat Model of PD

#### 3.1.1. CRSJ Can Improve the Behavioral Deficits in PD Rats

**Figure 1:**
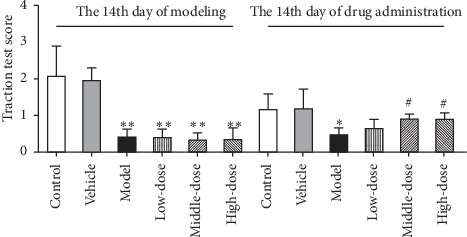
Traction test results in rats with or without exposure to the PD model and/or CRSJ administration. Compared with control group: ^*∗*^*P* < 0.05, ^*∗∗*^*P* < 0.01. Compared with model group: ^#^*P* < 0.05. PD, Parkinson's disease; CRSJ, Cong Rong Shu Jing.

Healthy rats have climbing abilities, which require proper grip strength and movement coordination. The traction test is a common observation model in Parkinson's disease to detect behavioral deficits in limb movement and coordination of rats [11], and we used it to detect the influence of CRSJ on the movement coordination in PD rats. The higher the score in the traction test, the longer the rats were able to hang from the wire, thus the stronger the grip of the rats. As shown in Figure 1, the test scores of the model and all CRSJ groups were significantly reduced on the 14^th^ day of the modeling (*P* < 0.01). After 14 days of CRSJ administration, the suspension time was prolonged in the medium- and high-dose groups (*P* < 0.05). The suspension scores of rats in the control and the vehicle groups were similar, and the difference between these groups was not statistically significant (*P* > 0.05). These results demonstrate that the administration of CRSJ can significantly improve the motor coordination in PD rats.

#### 3.1.2. CRSJ Can Increase the Neuronal TH Expression in the Substantia Nigra, as well as the Striatal DA Content, of PD Rats

**Figure 2:**
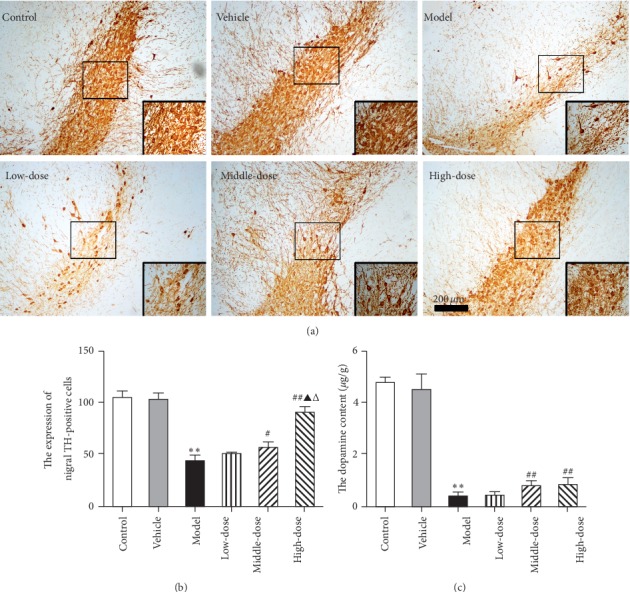
TH-positive cells in the SN and the striatal DA content of rats with or without exposure to the PD model and/or CRSJ treatment. (a) Immunohistochemistry showing TH-positive cells in the rat substantia nigra of different experimental groups (100× and 400×). (b) Quantitative analysis of TH-positive cells in the SN of rats. (c) The DA content in the striatum measured by HPLC. Compared with control group: ^*∗∗*^*P* < 0.01. Compared with model group: ^#^*P* < 0.05, ^##^*P* < 0.01. Compared with low-dose group: Δ*P* < 0.01. Compared with medium-dose group: Δ*P* < 0.01. SN, substantia nigra; DA, dopamine; PD, Parkinson's disease; CRSJ, Cong Rong Shu Jing; TH, tyrosine hydroxylase; HPLC, high-performance liquid chromatography.

TH is the rate-limiting enzyme in DA synthesis and is mainly present in DA neurons of the substantia nigra (SN). Decreased TH expression and activity in the SN and the resulting striatal DA deficiency are the main causes of PD [12]. In order to observe the effects of CRSJ on the presence of dopaminergic neurons and the striatal DA release in PD model rats, we used immunohistochemistry to detect the number of TH-positive cells in the SN of rats in each group and HPLC to determine the striatal DA content. In the control and vehicle groups, the SN specimens contained large numbers of TH-immunopositive neurons in orderly arrangement, the cell body was full, conical, or oval, and the neuronal processes were clearly demarcated (Figure 2(a)). In the model group, the number of TH-positive cells in the SN was significantly reduced (*P* < 0.01), the somata of the neurons appeared wrinkled, their contours and protuberances were often not clearly identifiable, and the DA content in the striatum was significantly reduced (*P* < 0.01; Figure 2(a)–2(c)). After CRSJ injection, the loss of TH-positive cells in the SN was reduced in the medium- and high-dose groups (*P* < 0.05 or *P* < 0.01), the neuronal shrinkage was partially prevented, and the striatal DA content was increased (*P* < 0.01; Figure 2(a)–2(c)). These results suggest that CRSJ can reduce the loss of dopaminergic neurons in the SN caused by rotenone and increase the release of DA in the striatum.

### 3.2. CRSJ Can Relieve ERS in PD Rats

#### 3.2.1. CRSJ Can Reduce *α*-Synuclein Expression in the Substantia Nigra of PD Rats

**Figure 3:**
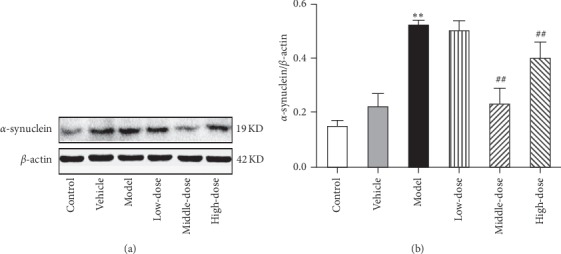
Expression of *α*-synuclein in the substantia nigra of rats. (a) Representative example of a Western blot. (b) Quantitative Western blot analysis of *α*-synuclein expression in rat midbrains of different experimental groups. Compared with control group: ^*∗∗*^*P* < 0.01. Compared with model group: ^##^*P* < 0.01.

The accumulation of abnormal proteins can cause ERS, and *α*-synuclein represents a large percentage of un- or misfolded proteins. To determine whether CRSJ administration can reverse the accumulation of *α*-synuclein, we assessed the protein content of *α*-synuclein in the midbrain of rats from each group. As shown in Figure 3, we found that the levels of *α*-synuclein were increased after rotenone administration (*P* < 0.01), whereas these levels were significantly decreased in the medium- and high-dose groups after the CRSJ intervention compared with the model group (*P* < 0.01). Thus, in the brain of PD rats, CRSJ administration can reverse the abnormal protein accumulation caused by rotenone.

#### 3.2.2. CRSJ Can Reduce the Expression Levels of ERS Pathway-Related Proteins in PD Rats

**Figure 4:**
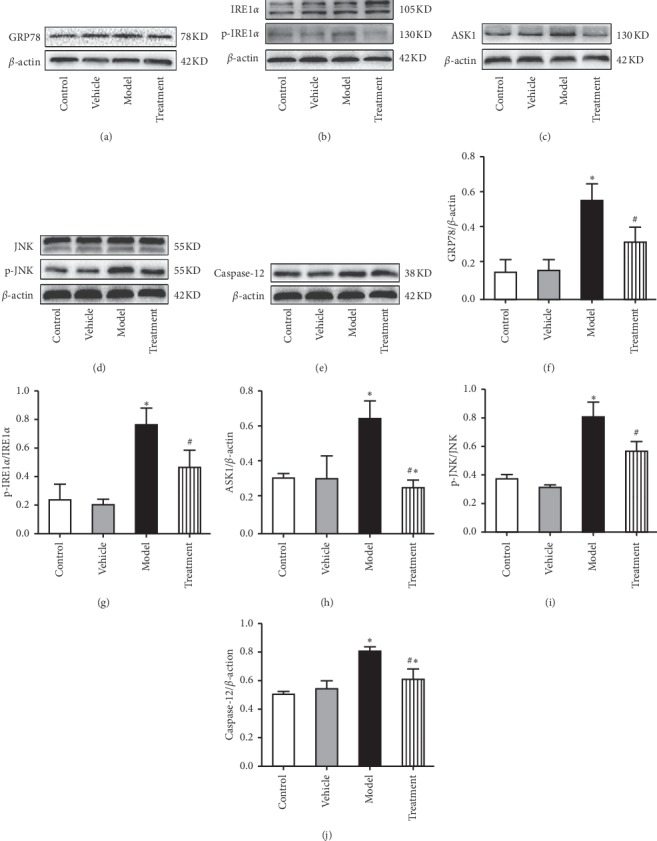
Expression of ERS pathway-related proteins in the substantia nigra of rats detected by Western blots. (a–e) Representative Western blots of GRP78 (a), p-IRE1*α* (b), ASK1 (c), p-JNK (d), and caspase-12 (e) protein expression in different experimental groups. (f–j) Quantitative analysis of GRP78 (f), p-IRE1*α* (g), ASK1 (h), p-JNK (i), and caspase-12 (j) protein levels in the midbrain substantia nigra of rats. Compared with control group: ^*∗*^*P* < 0.05. Compared with model group: ^#^*P* < 0.05. ERS, endoplasmic reticulum stress; GRP78, glucose regulatory protein 78; p-IRE1*α*, phosphorylated inositol-requiring enzyme 1; ASK1, apoptosis signal-regulating kinase 1; p-JNK, phosphorylated c-Jun N-terminal kinase.

According to Figure 3, the levels of abnormal *α*-synuclein were increased in the brain of PD rats after rotenone exposure. To examine whether the increase in *α*-synuclein was related to ERS generation and whether this rotenone-induced ERS was alleviated by the CRSJ intervention, the expression levels of ERS pathway-related proteins were detected in rat brains from all experimental groups. As shown in Figure 4, the expression levels of GRP78, p-IRE1*α*, ASK1, p-JNK, and caspase-12 proteins were increased in tissue samples of the model group compared with those of the control group (*P* < 0.05). Compared with the expression levels in the model group, the levels of the above-mentioned proteins in the CRSJ group were decreased (*P* < 0.05). These results suggest that CRSJ may alleviate rotenone-induced neuronal apoptosis by attenuating endoplasmic reticulum stress involving the GRP78-IRE1*α*-ASK1-JNK signaling pathway.

### 3.3. CRSJ Can Alleviate ERS by Increasing the Content of Neurotrophic Factors and Activating the PI3K/AKT Pathway

#### 3.3.1. CRSJ Can Increase the Expression of Neurotrophic Factors in the Brain of PD Rats

**Figure 5:**
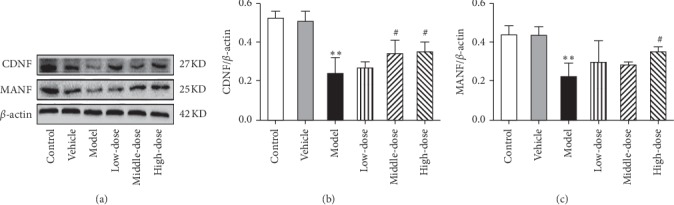
CDNF and MANF protein expression levels in the substantia nigra of rats of different experimental groups. (a) Western blot of CDNF and MANF protein expression levels in the substantia nigra of rats. (b-c) Quantitative Western blot analysis of CDNF (b) and MANF (c) protein levels in the substantia nigra. Compared with control group: ^*∗∗*^*P* < 0.01. Compared with model group: ^#^*P* < 0.05. CDNF, cerebral dopamine neurotrophic factor; MANF, mesencephalic astrocyte-derived neurotrophic factor.

Previous studies have shown that CRSJ treatment exerts in PD rats therapeutic effects that are associated with reduced ERS levels. Thus, we further elucidated the specific mechanisms of relieving ERS by CRSJ administration. First, the levels of the neurotrophic factors CDNF and MANF were measured in brain samples of rats from each group. As shown in Figure 5, we observed a decreased CDNF and MANF protein expression in the model group compared with the control and the vehicle groups (*P* < 0.05 or *P* < 0.01). Compared with the model group, the CDNF expression was increased in the CRSJ-treated medium- and high-dose groups (*P* < 0.05), whereas the MANF expression was only increased in the high-dose group (*P* < 0.05) with no statistically significant differences among the low-, medium-, and high-dose groups (*P* > 0.05).

#### 3.3.2. CRSJ Can Increase the Protein Expression Levels Related to the PI3K/AKT Pathway in PD Rats

**Figure 6:**
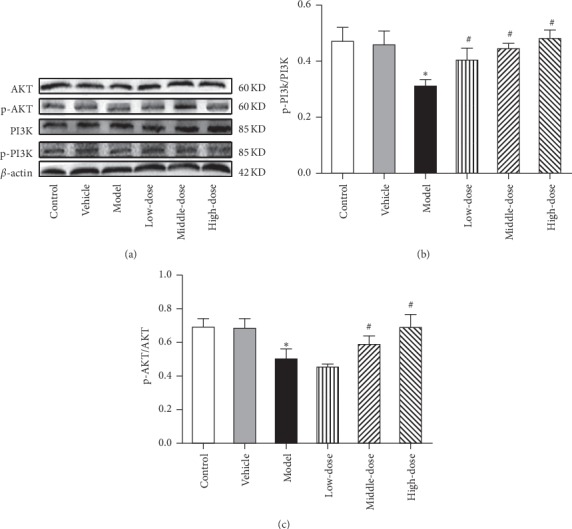
Expression of PI3K, AKT, p-PI3K, and p-AKT in the substantia nigra of animals from different experimental groups. (a) Western blot demonstrating the expression levels of PI3K, AKT, p-PI3K, and p-AKT in the substantia nigra of each experimental group. (b-c) Quantitative analysis of p-PI3K (b) and p-AKT (c) proteins in the substantia nigra of each group of rats. Compared with control group: ^*∗*^*P* < 0.05. Compared with model group: ^#^*P* < 0.05. PI3K, phosphoinositide 3-kinase; AKT, protein kinase B; p, phosphorylated.

As it has been reported that neurotrophic factors can activate the PI3K/AKT pathway and reduce ERS, the expression levels of PI3K/AKT pathway-related proteins in the midbrain of rats were detected in each group to further explore possible CRSJ targets that mitigate ERS. As shown in Figure 6, the expression levels of p-PI3K and p-AKT in the model group were reduced compared to those in the control group (*P* < 0.05). After the CRSJ intervention, the ratios of p-PI3K/PI3K protein expression levels were significantly increased in the low-, medium-, and high-dose groups compared to the model group (*P* < 0.05). Similarly, the ratios of p-AKT/AKT protein expression were significantly higher in the medium- and high-dose groups (*P* < 0.05). The results displayed in Figures 5 and 6 suggest that the ERS-relieving effects of CRSJ may be related to the elevated expression levels of neurotrophic factors in the brain activating the PI3K/AKT pathway.

## 4. Discussion

Multiple studies have shown that ERS is involved in the occurrence and development of PD [13, 14]. GRP78 is an important molecular chaperone protein of the endoplasmic reticulum. Physiologically, GRP78 binds to IRE1*α*, one of the transmembrane proteins of the endoplasmic reticulum. When a large amount of misfolded *α*-synuclein accumulates, ERS will be induced, leading to the dissociation of GRP78 from IRE1*α* and the subsequent activation of IRE1*α* through autophosphorylation or dimerization. The activated IRE1*α* can then bind to the TNF receptor-associated factor 2 (TRAF2), and this complex activates ASK1 to assemble the IRE1*α*/TRAF2/ASK1 trimer. ASK1 is part of the mitogen-activated protein kinase pathway that can prompt the downstream phosphorylation and activation of JNK. JNK is an important member of the family of mitogen-activated protein kinases, and phosphorylation of JNK can activate the ERS apoptosis pathway involving caspase-12 that induces programmed cell death [2].

Neurotrophic factors facilitate nerve cell growth and differentiation. Being highly homologous in their amino acid sequence, CDNF and MANF belong to the conserved family of dopamine neurotrophic factors, and they are located in the endoplasmic reticulum to counteract apoptosis induced by ERS. Research has shown that, in a rat model of Parkinson's disease, neuronal CDNF and MANF protect dopaminergic neurons and support nerve repair [15]. PI3K/AKT, which is widely distributed in the nervous system, is a classical signaling pathway regulating cell proliferation, differentiation, apoptosis, and aging. Its activation can be determined by the phosphorylation level of PI3K and AKT. The activation of this pathway can regulate apoptosis-related proteins downstream, thus inhibiting neuronal apoptosis [16]. Studies have shown that neurotrophic factors can activate the PI3K/AKT pathway and inhibit endoplasmic reticulum stress to reduce apoptosis [2].

In traditional Chinese medicine, PD belongs to the category of “fibrillation syndromes”. According to traditional Chinese medicine theory, this disease is probably caused by liver and kidney deficiencies in middle-aged and elderly patients and the emptiness of the marrow sea, which cannot help nourishing their limbs. Therefore, “nourishing the liver and kidney, filling the marrow, soothing the liver and muscles, eliminating wind, and preventing fibrillation” is an important treatment idea [17]. The CRSJ compound represents the summary of our research group's long-standing experience. This compound is composed of *Cistanche* spp., *Polygonatum* spp., *S. miltiorrhiza*, red peony root, and peony skin. Huangjing, which belongs to the *Polygonatum* family, acts as a minister medicine, exerts flat effects, has a sweet taste, and acts on the three meridians of spleen, kidney, and lung. It has the effect of tonifying the kidney and essence, nourishing the Yin, and moistening dryness. *S. miltiorrhiza*, red peony root, and peony skin are adjuvant medicines, which can remove blood stasis, relieve pain, and activate blood circulation through menstruation [18]. In previous clinical and basic research studies, our group found that the main CRSJ component, *C. deserticola*, can improve the UPDRS score of patients with PD, improve the behavioral scores of animals in PD models, increase the expression of glial cell line-derived neurotrophic factor and its receptor in ME23.5 cells, increase the expression of PI3K and AKT in PD model rats, and reduce the apoptosis of nerve cells [3–6]. However, the protective effect of the CRSJ compound on PD rats has not been studied before.

In the present study, rotenone was subcutaneously injected into the back of the neck to generate the PD model. Because this model selectively causes the degeneration and death of dopaminergic neurons in the substantia nigra and the formation of intracytoplasmic Lewy bodies, it can better than other PD models simulate the behavior and pathological characteristics of PD [19].

After two weeks of rotenone administration, the traction test scores in rats of the model group, the numbers of TH-positive cells in the brain, and the DA levels in the striatum were decreased, indicating that rotenone induced the core characteristics of PD in rats. This confirms the successful establishment of the PD model. After the CRSJ intervention, the scores of PD rats in the traction test were increased, the survival of dopaminergic neurons in the SN was improved, and the DA release in the striatum was enhanced, reflecting the protective effects of CRSJ treatment in PD rats.

In subsequent experiments, we found that the expression levels of endoplasmic reticulum stress pathway-related proteins, such as *α*-synuclein, GRP78, p-IRE1*α*, ASK1, p-JNK, and caspase-12, were increased in the rat midbrain of the model group, suggesting the presence of ERS in PD animals. This conclusion is in agreement with the results of Zhang et al. [20] and Qu et al. [21]. After CRSJ intervention, the expression levels of the above-mentioned indicator proteins were decreased in PD rats, indicating that CRSJ administration can effectively inhibit the activity of ERS-related proteins and protect nerve cells from apoptosis.

Finally, to observe whether the CRSJ-induced mechanisms that prevent ERS are related to increased expression of neurotrophic factors and activation of the PI3K/AKT pathway, the expression levels of CDNF, MANF, p-PI3K, and p-AKT were determined in the substantia nigra of rats. The results showed that after CRSJ administration to PD rats, the expression of neurotrophic factors, as well as the expression of p-PI3K and p-AKT, was increased. This suggests that CRSJ may upregulate the expression of neurotrophic factors, activate the PI3K/AKT pathway, and mitigate the ERS effects in PD rats.

Among Western drugs for PD treatment, levodopa formulations are mainly used. Their short-term potency is obvious, but the long-term application does not only weaken their efficacy but also causes obvious adverse reactions. By contrast, traditional Chinese medicine has certain advantages in improving the movement symptoms of PD, delaying the progress of the disease, reducing the adverse reactions of drugs, and improving the quality of life in PD patients [22].

Because the mechanisms of degenerative diseases in neurons are very complex and the conclusions of our animal experiments are mostly at a descriptive level, the causal relationships between up- and downstream ERS pathways and their fine adjustments have yet to be fully explored. In the future, cell experiments should be the focus to further explore the regulatory mechanisms between ERS-related proteins. This may provide new targets for better control of PD symptoms and progression.

## Data Availability

The data used to support the findings of this study are included within the article.
